# Phylogenetic analysis highlights the role of older people in the transmission of HIV-1 in Fuyang, Anhui Province, China

**DOI:** 10.1186/s12879-019-4187-9

**Published:** 2019-06-27

**Authors:** Jianjun Wu, Yu Zhang, Yuelan Shen, Xiaolin Wang, Hui Xing, Xiaohui Yang, Xinping Ding, Bing Hu, Hanping Li, Jingwan Han, Jingyun Li, Bin Su, Yongjian Liu, Lin Li

**Affiliations:** 1 Anhui Provincial Center for Disease Control and Prevention, Hefei, 230601 China; 2 Department of AIDS Research, State Key Laboratory of Pathogen and Biosecurity, Beijing Institute of Microbiology and Epidemiology, Beijing, 100071 China; 3 State Key Laboratory for Infectious Disease Prevention and Control, National Center for AIDS/STD Control and Prevention, Chinese Center for Disease Control and Prevention, Beijing, 102206 China; 4 Fuyang Center for Disease Control and Prevention, Fuyang, 236069 China

**Keywords:** HIV-1, Older people, Transmission clusters, Phylogenetic, Epidemiology

## Abstract

**Background:**

The proportion of older HIV-1 infected people in China has increased rapidly in recent years. Elucidation of the transmission characteristics of this high-risk population subgroup is helpful for the development of tailored interventions.

**Methods:**

A phylogenetic analysis was performed that uses available HIV-1 *pol* sequences amplified with nested RT-PCR from plasma samples of all newly diagnosed participants spanning from October 2017 to September 2018 in Fuyang, Anhui Province. Transmission clusters were identified as two or more sequences that shared a corresponding node with an aLRT-SH value ≥90 in the maximum-likelihood phylogenetic tree and had an overall mean genetic distance of ≤1.5%. A local transmission cluster was defined as a cluster that had more than 80% of its sequences from Fuyang. The role of older people in local HIV-1 transmission was determined using an integration of molecular and demographic data.

**Results:**

Of 362 available sequences, 14 subtypes, and 28 local transmission clusters were identified. It was found that the proportion of older people in the local transmission cluster (69/77, 89.61%) was much higher than that of younger people (46/114, 40.35%) (χ^2^ test, *P* < 0.001). In the pretreatment drug resistance analysis, the proportion of sequences with PDRMs in the local transmission cluster was not significantly different between the older people group (57.14%, 4/7) and non-old-aged group (11.11%, 1/9) (Fisher’s exact test, *P* > 0.05).

**Conclusion:**

By combining phylogenetic analyses with demographic data, more detailed information was provided about the local transmission structure in Fuyang. These findings suggested that older people play an important role in local transmission, and more tailored interventions for this population subgroup are urgently needed.

## Background

It has been commonly assumed that older people are not a high-risk group for human immunodeficiency viruses (HIV) infection [1]. However, when HIV has become a manageable chronic disease, older people living with HIV, defined as HIV-infected adults who are aged 50 and over, [2] hit a record high of approximately 5.8 million accounting for 15.8% of 36.7 million HIV infections in 2015, according to The Joint United Nations Program on HIV and AIDS (UNAIDS) report [3]. The report noted that older people also remained sexually active and exhibited a moderate to high level of interest toward sex well into their 80s [4–6]. Moreover, they were even more likely to have multiple partners and use condoms inconsistently [7]. These facts led to the realization that older people were becoming a high-risk group. Also, according to the Center for Disease Control and Prevention (CDC) annual data, the proportion of older people newly diagnosed with HIV-1 infection increased in recent years in some areas of China [8, 9].

Fuyang, neighboring Henan Province and located in the northwest of the Anhui Province, is an underdeveloped city with an area of 9775 km^2^ that represents this trend. According to a recent local HIV-1 epidemiologic survey, this city was once defined as an HIV-1 epidemic focus where infections have been primarily caused by commercial blood donations in the early phase of HIV-1 epidemics, [10, 11] and the number of newly reported HIV cases reached to its highest of 506 in 2004 due to the large-scale screening in previous blood donors. After implementing the policy of standardized management of blood stations, the reported HIV/AIDS cases decreased to its lowest of 143 in 2011. Meanwhile, the proportion of HIV infected through sexual contact was beginning to be higher than that of blood donations, and it has been determined that currently the HIV-1 epidemic is primarily driven by sexual transmissions, which accounted for more than 95%. By 2014, a total of 3521 HIV-1 infected cases (486 HIV infections, 1917 AIDS cases and 1118 deaths) had been reported, and the HIV prevalence was 0.031% (2403/7,823,000). Moreover, this survey also highlighted that the proportion of older newly HIV-1 infections rapidly increased from 19.0 to 32.1% in 2009–2014, though the proportion of local older people in Fuyang which had a residential population of 7.6–8.4 million has stabilized at 23% on average and didn’t change significantly in last decade. Elucidation of the transmission characteristics of this high-risk population subgroup is helpful for the development of tailored interventions.

Phylogenetic analysis is a useful tool that can be reliably used to define clusters of closely related sequences reflecting actual transmission, [12] while traditional epidemiological approaches based on behavioral information cannot. When combined with epidemiological and clinical data, more detailed transmission information can be identified that could be of public health relevance [13–15]. This method had been successfully used to implement tailored preventive interventions for different high-risk population subgroups, such as commercial sex workers, [16] men who have sex with men (MSM), [17] and injecting drug users (IDUs) [18].

The aim of this study is to reveal the local HIV-1 transmission structure of Fuyang by using HIV-1 *pol* sequences that have been proven to be sufficiently variable to permit the phylogenetic reconstruction of transmissions [19]. This study particularly focuses on older people with the goal of providing information regarding tailored preventive strategies.

## Methods

### Study population and specimens

All newly HIV-1 diagnoses in Fuyang from October 2017 to September 2018 were enrolled in this study. Blood samples were collected by the CDC of Fuyang for the purpose of baseline drug resistance genotyping. All 369 antiretroviral-naive participants signed written informed consents prior to sample donations, and 340 persons completed standardized questionnaires that included demographic data, while 29 persons denied. This study was reviewed and approved by the ethics committees of the Beijing Institute of Microbiology and Epidemiology and the Anhui Center for Disease Prevention and Control.

### Sequence data

Total viral RNA was extracted from plasma isolated from blood samples using the Roche High Pure Viral RNA kit (Roche, REF: 11858882001). A nested reverse transcriptase polymerase chain reaction (RT-PCR) was applied to amplify a *pol* fragment of the 1062 bp length region (from 2253 to 3314 according to HXB2 calibrator) spanning the protease gene and partial reverse transcriptase gene using the One Step RNA PCR Kit (Takara, RR055A) and the ExTaq Kit (Takara, RR902A) with sets of primers and thermal cycling conditions as described previously [20]. The PCR products were purified and subjected to direct DNA sequencing on an Applied Biosystems 3730 Sequencer.

### Phylogenetic analyses

Phylogenetic analysis was used to infer the HIV-1 subtypes. The sequences were then submitted to a quality control program on The Los Alamos National Laboratory (LANL) HIV database (https://www.hiv.lanl.gov) to check for laboratory contamination. Then, all the available sequences were aligned using reference strains of group M and the circulating recombinant forms (CRFs) in China using the ClustalW multiple alignment program included in the BioEdit software. They were then manually edited. A phylogenetic tree was constructed using the neighbor-joining (NJ) method based on the Kimura 2-parameter model with 1000 bootstrap replicates. For sequences of unknown HIV-1 subtype, the jumping profile hidden Markov model (jpHMM) (http://jphmm.gobics.de/submission_hiv) was applied to screen for recombination breakpoints.

For the purpose of transmission cluster analysis, we followed a methodology described previously and included a data set of similar sequences from LANL HIV database by identifying the ten closest matches with BLAST for each of the Fuyang sequences in the study [21]. The final data set containing 1645 sequences (*n* = 362 from Fuyang and *n* = 1283 from LANL sequences) was then aligned to find the best fitting nucleotide substitution model using the FindModel tool (https://www.hiv.lanl.gov). A maximum likelihood (ML) phylogenetic tree was constructed in the PhyML 3.0 online program using the GTR nucleotide substitution model and nearest neighbor interchanges (NNI) to estimate the tree topology by approximate likelihood ratio test Shimodaira-Hasegawa (aLRT-SH) like branch support [22]. In addition, the pairwise genetic distance was computed using the Tamura-Nei model in the MEGA6 software. Transmission clusters were picked out in ClusterPicker1.2 when the relationship among two or more sequences fulfilled the following two stringent conditions: the tree nodes with an aLRT-SH value ≥90 and an overall mean genetic distance less than 0.015 nucleotide substitutions per site [21, 23]. Clusters that had more than 80% sequences from Fuyang were defined as local transmission clusters [21].

### Pretreatment drug resistance analysis

For the analysis of drug resistance mutations (DRMs), all available sequences were submitted to the Genotypic Resistance Interpretation Algorithm of the Stanford drug resistance database (http://hivdb.stanford.edu). Pretreatment drug resistance mutations (PDRMs) were identified according to the WHO 2009 update of the DRMs list [24].

### Statistical analyses of demographic data

The demographic data were all based on a self-reported standardized questionnaire administered at the time of diagnosis. The variables of gender, age, education, marriage, transmission category, and HIV-1 subtype were evaluated for the purpose of depicting the local HIV-1 infection status. To further focus on elderly people, persons aged 50 or older were considered to be in the older people group, and persons younger than 50 years old were conversely classified into the non-old-aged group. All the variables that differed between these two groups were examined. Additionally, the contribution of older people to local HIV-1 transmission was also accessed by comparing the number of older people and non-old-aged people enrolled in local transmission cluster and non-local transmission cluster. All statistical analyses were tested using Pearson’s χ^2^ test or Fisher’s exact test based on a two-sided α of 0.05 using R version 3.5.1.

## Results

### Description of the demographic information

After excluding seven people due to the poor quality of their *pol* sequences, the final study population consisted of 333 people with demographic data available and 29 persons without demographic information.

Overall, the study population fraction of males to females was 4.29:1 (270/63). In reference to the transmission category, as many as 60.66% (202/333) of HIV-1 infected cases acquired the virus by heterosexual contact, 38.44% (128/333) of HIV-1 infected cases were MSM, 0.3% (1/333) were infected by injection drug use or heterosexual contact, 0.3% (1/333) were IDU, and 0.3% (1/333) of cases had an unknown transmission category. The median age of all of the participants was 44 years (Interquartile range: 31–59), of which the older people group accounted for 37.84% (126/333), and the proportion of persons in the non-old-aged group was 62.16% (207/333). The demographic comparison of these two groups is shown in Table 1. There was no significant difference in gender composition, but with regard to educated and married people, older people were more likely to be divorced (29.37% vs. 12.56%) and undereducated (the proportion of whom had a degree under high school: 92.06% vs. 68.08%). A more important fact is that the transmission category also exhibited statistically significant difference (*P* < 0.001). Older people were more likely to be infected through heterosexual contact (113/126, 89.68%), in contrast, the proportion of MSM (117/207, 56.52%) was higher than whom infected through heterosexual contact (89/207, 43.00%) in non-old aged group. Although there is the possibility of misclassification of MSM as heterosexuals and it has been confirmed in investigations conducted in UK, [25, 26] older people influenced by Chinese traditional culture especially in underdeveloped areas of China are more likely to be heterosexual, and it is also found that some of them are even involved in drug-used heterosexual activities [27].

**Table 1:** Demographic differences of older people group vs. non-old aged group with HIV-1 infection in Fuyang city

	Total^a^		Older people group (≥50 years old)		Non-old aged group (< 50 years old)		χ^2^ *P* value^#^
	n	%	n	%	n	%	
Sex							
Male	270	81.08	99	78.57	171	82.61	0.44
Female	63	18.92	27	21.43	36	17.39	
Transmission category							
Heterosexual contact	202	60.66	113	89.68	89	43.00	< 0.001
MSM	128	38.44	11	8.73	117	56.52	
IDU	1	0.30	1	0.79	0	0	
Heterosexual/IDU	1	0.30	0	0	1	0.48	
Unknown	1	0.30	1	0.79	0	0	
Education							
Illiteracy	45	13.51	40	31.75	5	2.41	< 0.001
Primary school	98	29.43	49	38.89	49	23.67	
Middle school	116	34.83	27	21.43	89	43.00	
High school	45	13.51	10	7.94	35	16.91	
College or bachelor above	29	8.71	0	0	29	14.01	
Marriage							
Married	195	58.56	78	61.90	117	56.52	< 0.001
Unmarried	74	22.22	11	8.73	63	30.43	
Divorced or Unmatched	63	18.92	37	29.37	26	12.56	
Unknown	1	0.30	0	0	1	0.48	

^a^Only 333 HIV-1 infected people with demographic date are shown

# All statistical analyses were tested with the Pearson’s χ^2^ test based on a two-sided of 0.05 using R version 3.5.1

### HIV-1 genotype distribution

**Fig. 1:**
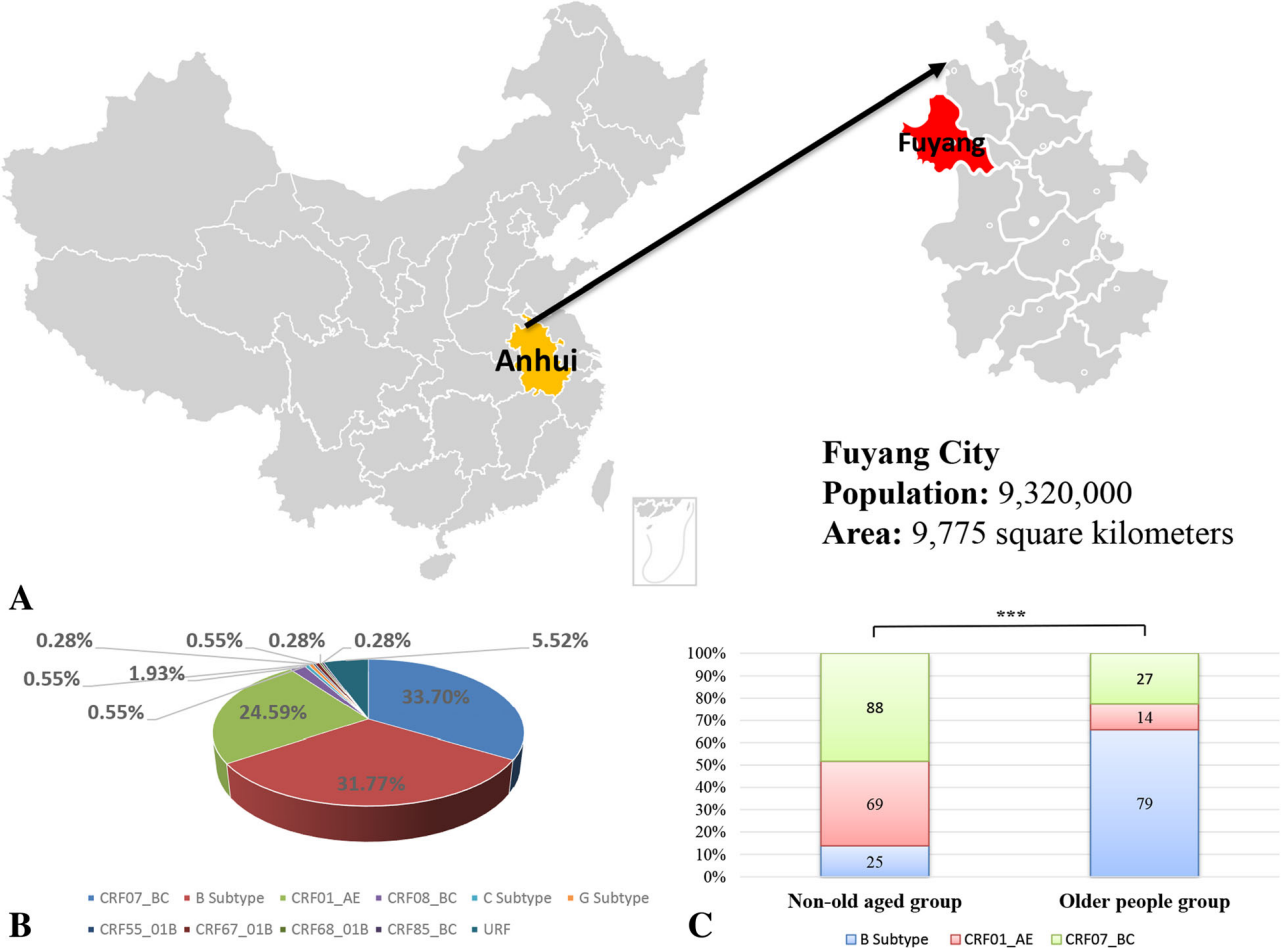
**a**. Map of China highlighting Fuyang, Anhui Province with the population and area labeled below. **b**. Pie chart in the lower-left corner representing the HIV-1 subtype distribution of the 362 newly diagnosed people, with available sequences from October 2017 to September 2018 in Fuyang. **c**. Differences in the constituent ratios of three major circulating subtypes (B subtype, CRF01_AE, and CRF07_BC) between the older people group and the non-old-aged group. “***” represents *P* < 0.001

Of the 362 available sequences, a total of 14 subtypes consisting of three pure subtypes, seven CRFs, and four unique recombinant forms (URFs) were identified. The distribution of HIV-1 subtypes was as follows: CRF07_BC (122/362, 33.70%), B (115/362, 31.77%), CRF01_AE (89/362, 24.59%), CRF08_BC (7/362, 1.93%), C (2/362, 0.55%), G (2/362, 0.55%), CRF55_01B (1/362, 0.28%), CRF67_01B (2/362, 0.55%), CRF68_01B (1/362, 0.28%), CRF85_BC (1/362, 0.28%), and URF (20/362, 5.52%) (Fig. 1b). CRF07_BC, B and CRF01_AE primarily contributed to the local HIV-1 epidemic. With respect to the constituent ratio of these three major circulating subtypes, the older people group was significantly different from the non-old-aged group (χ^2^ test, *P* < 0.001). Older people were primarily infected with the B subtype virus (62.70%, 79/126), followed by CRF07_BC (21.43%, 27/126) and CRF01_AE (11.11%, 14/126). However, CRF07_BC (42.51%, 88/207) dominated in the non-old-aged group, followed by CRF01_AE (33.33%, 69/207) and the B subtype (12.08%, 25/207) (Fig. 1c).

### Older people played an important role in local transmission

A total of 86 transmission clusters consisting 485 sequences (*n* = 207 from Fuyang and *n* = 278 reference sequences) was identified. Of them, 28 were local transmission clusters, with 14 clusters in the B subtype, 9 clusters in CRF07_BC, 2 clusters in CRF01_AE and 3 clusters in other subtypes. The distribution of non-local transmission clusters among subtypes was significantly different (χ^2^ test, P < 0.001), with 35 clusters in CRF01_AE, 16 clusters in CRF07_BC, 5 clusters in other subtypes and only 2 clusters in B subtype.

For the purpose of explicating the importance role of older people in local transmission, the proportion of older people and non-old-aged people in local transmission cluster was compared. With 207 people from Fuyang identified in transmission cluster, 77 were older people, 114 were non-old-aged people and 16 people with unknown age. It was found that the proportion of older people in local transmission cluster (69/77, 89.61%) was much higher than that of non-old-aged people (46/114, 40.35%) (χ^2^ test, P < 0.001).

**Fig. 2:**
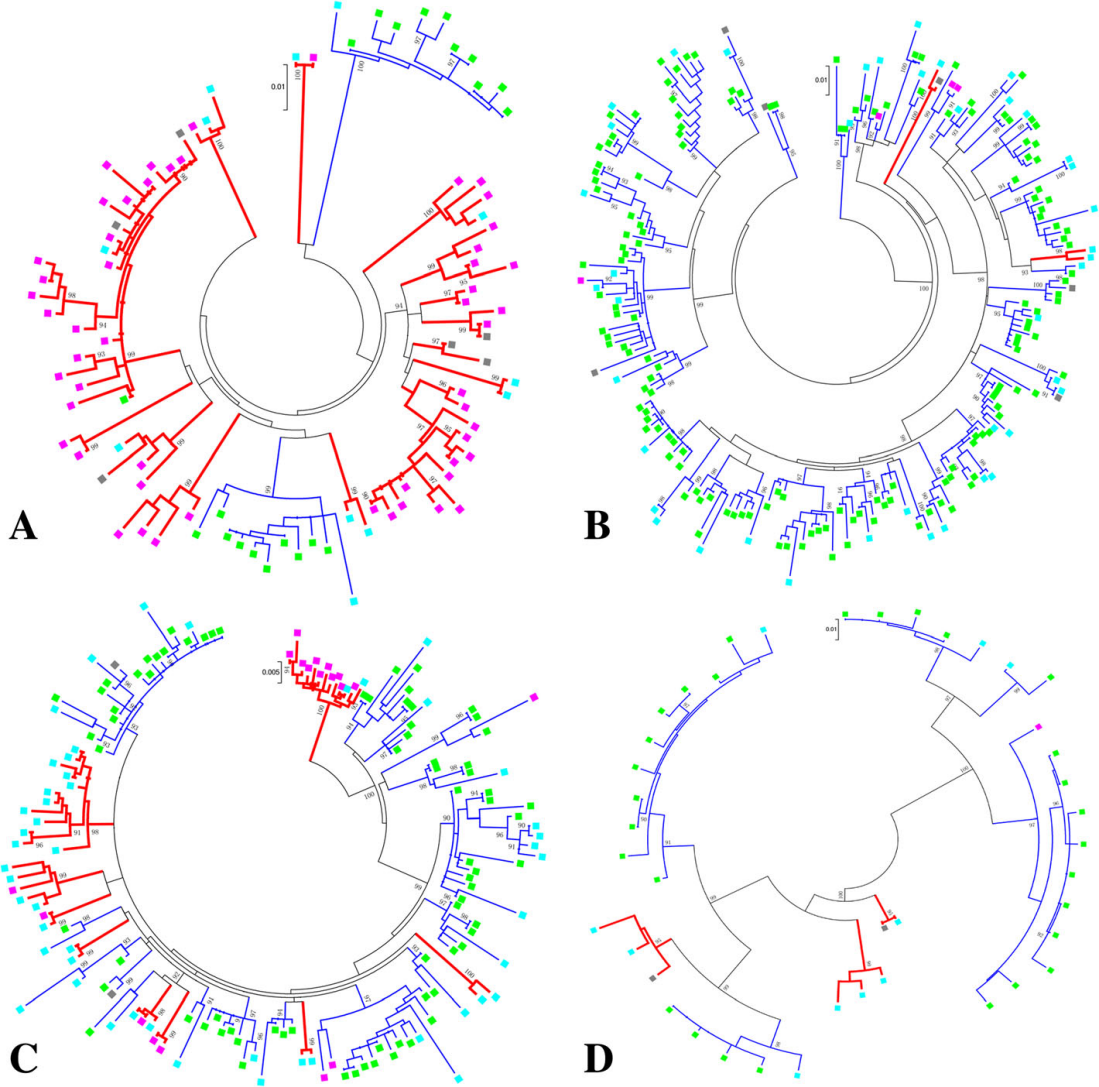
Transmission clusters in the maximum likelihood (ML) phylogenetic trees. A total of 86 transmission clusters consisting 485 sequences (*n* = 207 from Fuyang and *n* = 278 reference sequences) were identified by constructing the ML tree. The results are shown by subtype groups: **a**. B subtype, **b**. CRF01_AE, **c**. CRF07_BC and **d**. Other subtypes. The branch support (aLRT-SH) above 90% is shown at the corresponding nodes, and corresponding branch colors indicate cluster classifications (Red represents the local transmission cluster; Blue represents the non-local transmission cluster). Square colors indicate the sequences from different population subgroups (Pink: older people; Light blue: non-old-aged people; Light green: reference sequences; Grey: unknown-aged people)

Interestingly, more than half of older people in local transmission cluster (43/69, 62.32%) were identified in three large clusters (11, 12, and 22 sequences). Of these large clusters, one was all older people (13/13) and the other two clusters contained 84.62% (11/13) and 86.36% (19/22) of older people (Fig. 2). The median age of these clusters was homogeneous and is as follows: 73 years (13/13, Interquartile range: 69–77), 68 years (11/13, Interquartile range: 59–70), and 70 years (19/22, Interquartile range: 61.75–75). On the contrary, only one large local transmission cluster containing 10 non-old-aged people was identified. Taken together, these results suggested that older people played an important role in local transmission.

### Pretreatment drug resistance

The overall rate of PDRMs in this study population was 4.42% (16/362), which was as low as the percentage reported in a previous survey from the Anhui Province [20]. The rate was 4.35% (9/207) in non-old-aged group, while it reached 5.56% (7/126) among older people (Fisher’s exact test, *P* > 0.05). As shown in Table 2, all seven older people shared the K103 N mutation that confers resistance to nonnucleoside reverse transcriptase inhibitors (NNRTIs). In contrast, the PDRMs were more complicated among the nine non-old-aged people, and they had eight mutations that conferred resistance to NNRTIs, nucleoside reverse transcriptase inhibitors (NRTIs), and protease inhibitors (PIs). Moreover, the proportion of sequences with PDRMs in local transmission cluster was 57.14% (4/7) in older people group, while it was only 11.11% (1/9) in non-old-aged group (Fisher’s exact test, P > 0.05).

**Table 2:** All participants with pretreatment drug resistance mutations

Sample	Age	Subtype	Targeting drugs of resistant mutations		
			NRTIs	NNRTIs	PIs
076	58	B		K103 N	
098^a^	65	B		K103 N	
132^a^	52	B		K103 N	
134^a^	56	B		K103 N	
135^a^	56	B		K103 N	
322	71	01_AE		K103 N	
373	72	B		K103 N、K101E	
059	37	07_BC	M184 V		
063	47	01_AE		K103 N、L100I	
064	46	01_AE		K103 N、L100I	
166	43	07_BC	K219 N	Y181C	
173	43	01_AE			M46 L
231^a^	48	B		K103 N	
263	46	B		K103 N	
343	36	07_BC		V106 M	
345	34	08_BC		G190A	

*PI* protease inhibitors, *NRTI* nucleoside reverse transcriptase inhibitors, *NNRTI* non-nucleoside reverse transcriptase inhibitors; “^a^”, sequences in local transmission clusters

## Discussion

This analysis investigated the complicated HIV-1 epidemic in Fuyang that was identified to have 14 subtypes within 1 year. In contrast with the result of a nationwide molecular epidemiological survey indicating CRF01_AE was the most prevalent strain in China after 2007, [28] it was found that CRF07_BC predominated in this area, followed by subtype B that accounted for as much as 31.77% of the strains. It is rare to have such a high proportion of subtype B after the early-mid 1990s when commercial plasma collection in central China resulted in an explosion of subtype B infections among former plasma donors (FPDs) [29]. According to a recent local HIV-1 epidemiologic survey, the epidemic in Fuyang was once affected by FPDs. It is now primarily driven by sexual contact transmission [11]. Therefore, these results suggested that subtype B strains had possibly spread out of the FPDs into the general population through sexual contact. This hypothesis is consistent with the result of our previous study conducted in Henan Province [30].

With further phylogenetic analysis, subtype B sequences also showed a tendency to be in local transmission clusters. Moreover, of the 69 subtype B sequences in local transmission clusters, 72.46% (50/69) sequences were obtained from older people infected through heterosexual contact. When combined with clusters in CRF01_AE and CRF07_BC, a more significant result was found. This was that older people were more likely to be in local transmission cluster. In particular, only one reference sequence from LANL HIV database was included in three large clusters in total. On the contrary, non-old-aged people were more likely to be clustered with reference sequences obtained from other cities in China to form national transmission clusters. This suggested that older people predominated in the local HIV-1 transmission. This discrepancy could be attributed to social and economic factors. Fuyang is a relatively economically underdeveloped city compared to first-line cities, such as Beijing and Guangzhou, where transmission clusters among older people have rarely been identified [31, 32]. Older people in Fuyang prefer to settle down, while non-old-aged people tend to work outside the city. Statistically, about 2.8 million Fuyang residents were migrant workers [11]. The possibility cannot be excluded that some non-old-aged people were seasonal migrants who were infected outside Fuyang and were diagnosed locally. As a consequence, non-old-aged infected people might engage in multiregional virus exchange, while older people are more likely to contribute to local transmission. Future studies that track the floating population may help delineate the local epidemic more precisely.

A sexual behavior survey conducted in Guangxi, China, indicated that older people had little knowledge of HIV-1 preventions and did not perceive themselves to be at risk of HIV-1 [7]. Their lower education, which was found in this study, may be one of the major factors that contribute to the increasingly high proportion of older infected people and active transmission among this group. Moreover, another important factor often overlooked is the lack of emotional care, especially in older people who are divorced. A previous study found that older people were more vulnerable to loneliness and social isolation, [33] and some of them may negatively internalize or experience stigma when they are infected with HIV-1 [34]. As a result, they may engage in high-risk behaviors. This suggests that preventive interventions only are not adequate and more emotional and social support is also needed. Actually, we have not only taken actions to publicize the HIV knowledge by media like TV and radio, but also given lectures on HIV knowledge and humanistic care in communities and rural areas. By now, the treatment target is estimated to be achieved to “75–94-92”.

Though 94% of people with diagnosed HIV infection have received sustained antiretroviral therapy (ART), we should also focus on the increasing PDRMs due to extensive use of ART. In this study, the sequences of the older people had a prevalence of PDRMs (5.56%). Furthermore, all of the older people having PDRMs shared K103 N TDR site, and 51.95% (4/7) of them were in local transmission clusters. This implied that prevention from local transmission of K103 N which has high-level resistance to the free drugs (efavirenz and nevirapine) for HIV infections in China was in need, otherwise it will give rise to a reduction in efficacy of first-line ART. Added to this, older people were often associated with age-related chronic diseases, such as cardiovascular disease, cancer, hypertension, and diabetes. Such circumstances might raise the base-line treatment complexity and increase healthcare costs when coupled with PDRMs. Therefore, tailored preventive interventions for older people are urgently needed.

The generalizability of these results is subjected to certain limitations. For instance, although all newly diagnosed participants in Fuyang within 1 year were recruited, our results only show a representation of central cities in China. There are an increasing number of infected older people cases that have been reported for many other regions. Therefore, the contribution of older people to HIV-1 transmission should draw attention. The phylogenetic analysis applied in this study provides a useful tool for investigations of population-based HIV-1 transmission. However, our results are limited by the disadvantages of cross-sectional samples, which are unable to assess transmission dynamics. Therefore, longitudinal observational studies need to be conducted in the future to obtain more information regarding transmission among older people.

Despite these limitations, this is the first investigation to reveal the transmission structure among older people in China using an integration of molecular, clinical, and demographic data. These findings suggest that older people play an important role in local transmission, and as a previous survey suggested, limited knowledge about HIV-1 results in a lack of motivation to seek testing [35]. Therefore, the contribution of older people may be underestimated to some extent. Importantly, it is urgent that more tailored interventions be made and implemented. Otherwise, the treatment of older HIV-1 infected people will pose a great challenge in the future.

## Conclusions

This study highlighted that older people which was commonly assumed not to be a high-risk group for HIV-1 infection played an important role in the contribution of local HIV-1 transmission in Fuyang, Anhui Province, China. As the increasing numbers of older HIV-1 infected people were reported according to UNAIDS data, more attentions to older people should also be paid in other regions. By combining with demographic data, phylogenetic analysis was a useful tool can be applied to provide more tailored interventions which were definitely needed to the older people population subgroup.

## Data Availability

The gene sequences were deposited in the GenBank with the accession number: MK458938 – MK459299.

## Abbreviations

ART: Antiretroviral therapy
CDC: the Center for Disease Control and Prevention
CRFs: Circulating recombinant forms
DRMs: Drug resistance mutations
FPDs: Former plasma donors
HIV: Human immunodeficiency viruses
IDUs: Injecting drug users
jpHMM: the jumping profile hidden Markov model
LANL: Los Alamos National Laboratory
ML: Maximum likelihood
MSM: Men who have sex with men
NJ: Neighbor-joining
NNI: Nearest neighbor interchanges
NNRTIs: Nonnucleoside reverse transcriptase inhibitors
NRTIs: Nucleoside reverse transcriptase inhibitors
PDRMs: Pretreatment drug resistance mutations
PIs: Protease inhibitors
RT-PCR: Reverse transcriptase polymerase chain reaction
UNAIDS: the Joint United Nations Program on HIV and AIDS
URFs: Unique recombinant forms
